# Gene model for the ortholog of *CG31087* in *Drosophila arawakana*

**DOI:** 10.17912/micropub.biology.002315

**Published:** 2026-07-29

**Authors:** Taneille Jordan, Pablo Chialvo, Clare Scott Chialvo

**Affiliations:** 1 Biology, Appalachian State University, Boone, North Carolina USA

## Abstract

We developed a gene model for the *CG31087* ortholog in the ASM1815116v1 Genome Assembly (GenBank Accession: GCA_018151165.1 ) of *Drosophila arawakana* . This ortholog was characterized as part of a developing dataset for a comparative study of detoxification gene family evolution in the *immigrans* - *tripunctata* radiation of the genus *Drosophila* using an adapted Genomics Education Partnership gene annotation protocol for Course-based Undergraduate Research Experiences.

**Figure 1. Genomic neighborhood and gene model for CG31087 in D. arawakana:** (A) **Synteny comparison of the genomic neighborhoods for *CG31087* in *Drosophila melanogaster* and *Drosophila arawakana* .** Thin underlying arrows indicate which DNA strand the target gene, *CG31087* , is located on in *D. melanogaster* (top) and *D. arawakana* (bottom). The thin arrow pointing to the left indicates that *CG31087* is on the negative strand in *D. melanogaster* , and the thin arrow pointing to the right indicates that *CG31087* is on the positive strand in *D. arawakana* . The wide gene arrows pointing in the same direction as *CG31087* are on the same strand relative to the thin underlying arrows, while wide gene arrows pointing in the opposite direction of *CG31087* are on the opposite strand relative to the thin underlying arrows. White gene arrows in *D. arawakana* indicate orthology to the corresponding gene in *D. melanogaster* . Other colors of arrows indicate: black = non-orthology and grey = present in both neighborhoods but not syntenic. Gene symbols given in the *CG31087* gene arrows indicate the orthologous gene in *D. melanogaster* , while the locus identifiers are specific to *D. arawakana* . (B) **Gene Model in GEP UCSC Track Data Hub** (Raney et al., 2014). The coding-regions of *CG31087* in *D. arawakana* are displayed in the User Supplied Track (red); coding sequences (CDS) are depicted by thick rectangles and introns by thin lines with arrows indicating the direction of transcription. Subsequent evidence tracks include Spaln of *D. melanogaster* Proteins (purple, alignment of Ref-Seq proteins from *D. melanogaster* ), Coding Regions Predicted by Augustus (dark blue), GeMoMa (teal), and NSCAN PASA-EST (dark green), and RNA-Seq from mixed sex adult flies (brown; alignment of Illumina RNA-Seq reads from *D. arawakana* – Erlenbach et al., 2023). (C) **Dot Plot of CG31087-PA in *D. melanogaster* ( *x* -axis) vs. the orthologous peptide in *D. arawakana* ( *y* -axis).** Amino acid number is indicated along the left and bottom; CDS number is indicated along the top and right, and CDSs are also highlighted with alternating colors. In *D. melanogaster* , CG31087-PA is composed of five CDSs. However, the third and fourth CDS in *D. melanogaster* occur as a single CDS (CDS 3) in *D. arawakana* . Line breaks in the dot plot indicate areas of with low sequence identity between species. There is one longer break in CDS 1 (dark purple box – a) and one short break in CDS 4 (light blue box – b). (D) **Idiosyncrasies in protein alignment.** We noted breaks in the protein alignments that indicate low levels of sequence similarity. In CDS 1, there is a long break (dark purple box – a) that spans the first 57 amino acids of the ortholog. Only five of these amino acids are dissimilar and there are also three deletions. In CDS 4, there is a short break (light blue box – b) that spans a region of 24 amino acids (370-393). Eighteen of the amino acids are similar, and only four are dissimilar.

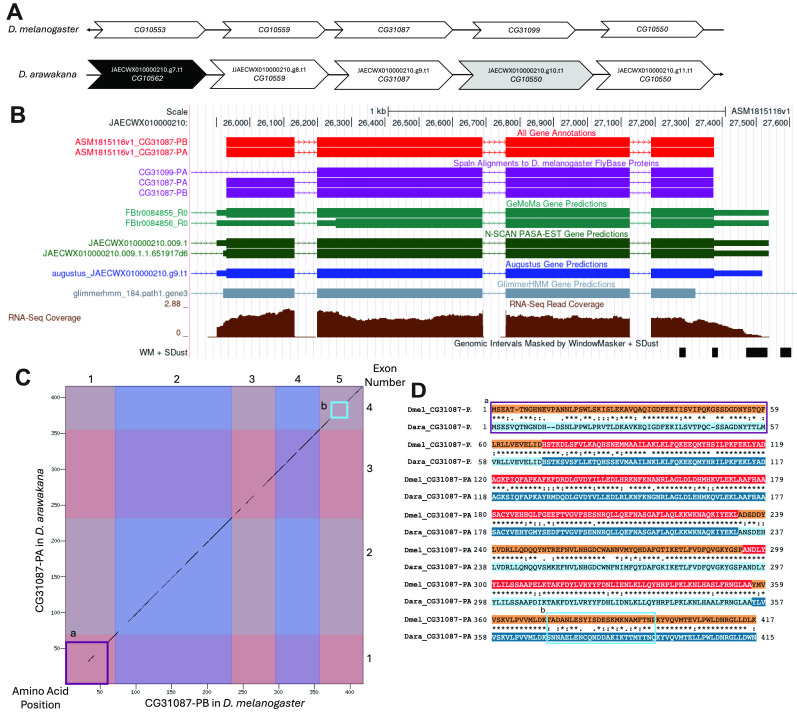



## Description

**Table:** 

*This article reports a predicted gene model generated by undergraduate work using a structured gene model annotation protocol defined by the Genomics Education Partnership (GEP; thegep.org) for Course-based Undergraduate Research Experience (CURE). The following information in quotes may be repeated in other articles submitted by participants using the same GEP CURE protocol for annotating Drosophila species orthologs of Drosophila melanogaster detoxification genes.* “Within insects, the process of detoxifying xenobiotics and host secondary metabolites is a three-phase process that involves functionalization, conjugation, and excretion of these compounds. Expansions of known detoxification gene families ( *e.g.* , cytochrome P450s) is associated with diet breadth and insecticide resistance (Ranson et al., 2002; Després et al., 2007; Rane et al., 2016). With the increasing availability of high-quality genomes for non-model organisms, including *Drosophila* species beyond *D. melanogaster* , it is now possible to perform large scale comparative studies (Robinson et al., 2011; Kim et al., 2021; Threfall and Baxter, 2021). Careful manual annotation and curation of gene models can improve upon computational gene predictions in non-model species, which aids the accuracy of studies on gene and genome evolution (Mudge and Harrow, 2016; Tello-Ruiz et al., 2019). To aid in these annotations, the Genomics Education Partnership (thegep.org) developed a curriculum involving web-based tools that allow undergraduates to engage in authentic course-based research focused on manually annotating genes in non-model species (Rele et al., 2023). The orthologous gene models, including the one presented here, then provide a reliable basis for further evolutionary genomic analyses when made available to the scientific community. The gene ortholog described here in *Drosophila arawakana* for *CG31087* , a member of the ecdysteroid kinase-like gene family, was characterized as part of a developing dataset for a comparative study of detoxification gene families in the *immigrans* - *tripunctata* radiation of the genus *Drosophila* .” (Williams et al., 2026) Within the subgenus *Drosophila* , *D. arawakana* Heed 1962 is a member of the *dunni* subgroup in the *cardini* species group of the *immigrans-tripunctata* radiation (Heed and Krishnamurthy, 1959; Bächli, 2005). Species in the *dunni* subgroup, including *D. arawakana* , are distributed across the Caribbean (Heed, 1962). Members of the *cardini* group primarily feed and develop on fruit and flowers (Markow and O'Grady, 2008). While some mushroom-feeding *cardini* subgroup members tolerate the cyclopeptide toxin α-amanitin (Stump et al., 2011), the fruit/flower feeding species in the *dunni* subgroup do not (Erlenbach et al., 2023). “The ecdysteroid kinase-like genes (EcKL) are classified as arthropod specific phase II detoxification enzymes (Blum et al., 2020; Scanlan et al., 2020). These gene act by phosphorylating both hormones associated with insect metamorphosis and xenobiotics (Sonobe et al., 2006; Scanlan and Robin, 2024). Although detoxifiying compounds through the addition of phosphates is rare in mammals, this method is common in insects and bacteria (Mitchell, 2015; Scanlan et al., 2022).” (Canard et al., 2026)

*CG31087* is an ECKL gene that shows very high levels of expression in the midgut of fly larvae and adults of both sexes (Robinson et al., 2012; Leader et al., 2018). Expression of *CG31087* is induced by the *Cap ‘n' Collar* gene, which is a central regulator of xenobiotic detoxification responses (Misra et al., 2011). In a study to identify detoxification genes whose expression increased following exposure to xenobiotics, *CG31087* received a score of two (Scanlan et al., 2020).

We propose a gene model for the *D. arawakana* ortholog of the *D. melanogaster* *CG31087* gene. The genomic region of the ortholog corresponds to the Augustus gene prediction JAECWX010000210.g9.t1 in the ASM1815116v1 Genome Assembly of *D. arawakana* ( GCA_018151165.1 – Kim et al., 2021). This model is based on mixed sex, adult RNA-Seq data from *D. arawakana* (Erlenbach et al., 2023; https://doi.org/10.5061/dryad.hdr7sqvq2) and *CG31087* in *D. melanogaster* using FlyBase release FB2024_02 ( GCA_000001215.4 ; Gramates et al., 2022; Jenkins et al., 2022; Larkin et al., 2021).

***Synteny***

The reference gene, *CG31087 ,* occurs on chromosome 3R in *D. melanogaster* and is flanked upstream by *CG10559* and *CG10553* and downstream by *CG31099* and *CG10550* . The *tblastn* search of *D. melanogaster* CG31087-PA (query) against the *D. arawakana* (GenBank Accession: GCA_018151165.1 Genome Assembly (ASM1815116v1)) placed the putative ortholog of *CG31087* within contig_220 (JAECWX010000210.1) which corresponds to the Augustus gene prediction JAECWX010000210.g9.t1 (E-value: 0.0; percent identity: 78.61% as determined by *blastp* ). The putative ortholog is flanked upstream by the Augustus gene predictions JAECWX010000210.g8.t1 and JAECWX010000210.g7.t1, which correspond to *CG10559* and *CG10562* in *D. melanogaster* (E-value: 0.0 and 3.00e-141; identity: 65.38% and 49.02%, respectively, as determined by *blastp* ; Figure 1A ; Altschul et al., 1990). The putative ortholog of *CG31087* is flanked downstream by the Augustus gene predictions JAECWX010000210.g10.t1 and JAECWX010000210.g11.t1, which both correspond to *CG10550* in *D. melanogaster* (E-value: 0.0 and 0.0; identity: 65.57% and 63.68%, respectively, as determined by *blastp* ). The putative ortholog assignment for *CG31087* in *D. arawakana* is supported by the following evidence: The gene predictions surrounding the *CG31087* ortholog are mostly orthologous to the genes at the same locus in *D. melanogaster* and gene expression data corresponds with each prediction. The gene predictions are supported by E-values and percent identities. In the syntenic neighborhood, the gene prediction that corresponds to the second upstream gene was identified as the fourth upstream gene in *D. melanogaster* . Downstream of the target gene, both predictions correspond to *CG10550* , which is the second downstream gene in *D. melanogaster* . Scanlan et al. (2020) identified this gene as being a member of a clade that is expanding (four duplications identified). Thus, we conclude that the Augustus gene prediction JAECWX010000210.g9.t1 represents an ortholog of *CG31087* in *D. arawakana* ( Figure 1A ).

***Protein Model***

*CG31087* in *D. arawakana* has four coding sequences (CDS) within the genome sequence, and it encodes one unique protein sequence. The only unique protein sequence is translated from two messenger RNAs that differ in their untranslated regions (CG31087-PA and CG31087-PB; Figure 1B ). Relative to the ortholog in *D. melanogaster* , the CDS number is not conserved, but the protein isoform count is conserved. The *D. arawakana* ortholog of *CG31087* has one fewer CDS than *D. melanogaster.* We can see that the third and fourth CDS in *D. melanogaster* are a single CDS (CDS 3) in *D. arawakana* ( Figure 1D ) *.* The sequence of CG31087-PA in *D. arawakana* has 78.2% identity (90.4% similarity) with the protein-coding isoform CG31087-PA in *D. melanogaster* , as determined by *blastp* ( Figure 1C ). This level of divergence is not surprising given that *D. arawakana* and *D. melanogaster* belong to two separate subgenera ( *Drosophila* and *Sophophora* respectively) that diverged approximately 45-60 MYA (Russo et al., 1995; Tamura et al., 2004; Obbard et al., 2012). Coordinates of this curated gene model are archived in the CaltechDATA repository (see “Extended Data” section below).

## Methods

The annotation methods used in this project are adapted from those described in Rele et al. (2023), which includes algorithms, database versions, and citations for the complete annotation process developed for the Pathways Project. The methods for the current project are detailed in brief below with notes on significant differences between this protocol and the one described in Rele et al. (2023). The students use the GEP instance of the UCSC Genome Browser v.435 (https://gander.wustl.edu ; Kent et al., 2002; Raney et al., 2024) to examine the genomic neighborhood of their reference detoxification gene in the *D. melanogaster* genome assembly (Aug. 2014; BDGP Release 6 + ISO1 MT/dm6). Students obtain the protein sequence for the *D. melanogaster* target gene for a given isoform and use a *tblastn* search of the sequence against their target *Drosophila* species genome assembly ( *D. arawakana* ( GCA_018151165.1 – Kim et al., 2021)) on the NCBI BLAST server (https://blast.ncbi.nlm.nih.gov/Blast.cgi, Altschul et al., 1990) to identify the putative ortholog location. Students compare the genomic neighborhood of the putative ortholog to that of the reference gene in *D. melanogaster* . This local synteny analysis includes a minimum of two upstream and downstream genes relative to the potential ortholog. As no RefSeq protein data is available for these species, comparisons are based on gene predictions that correlate with gene expression data in the putative ortholog neighborhood. Using the multiple alignment tracks feature in the Genome Browser, students examine other sets of genomic evidence, including Spaln alignment of *D. melanogaster* proteins, multiple gene prediction tracks (e.g., GeMoMa, Augustus, NSCAN PASA-EST), and mixed sex RNA-Seq adult expression data from the target species generated by Erlenbach et al. (2023; https://doi.org/10.5061/dryad.hdr7sqvq2). Information on the genomic structure information (e.g., CDSs, intron-exon number, number of isoforms) for the reference gene in *D. melanogaster* is retrieved using Gene Record Finder (https://gander.wustl.edu/~wilson/dmelgenerecord/index.html; Rele et al *.,* 2023). To determine approximate splice sites within the target gene, a *tblastn* search using the CDSs from the *D. melanogaste* r reference gene against the putative ortholog location (10kb up- and downstream of the target gene prediction). Coordinates of the CDS(s) are refined by examining aligned RNA-Seq data, identifying canonical splice site sequences, and ensuring the maintenance of an open reading frame. Students confirm the biological validity of their target gene model using the FlySeq Gene Model Checker (https://gander2.wustl.edu/~wilson/genechecker-flyseq/), which compares the hypothesized target gene model's structure and translated sequence against the *D. melanogaster* reference gene. At least two independent models for this gene are generated. These models are reconciled by a third independent researcher to produce the final model presented here. Note: comparison of 5' and 3' UTR sequence information is not included in this GEP CURE protocol.

## Data Availability

Description: Zipped archive containing FASTA, PEP, and GFF files for the CG31087 model. Resource Type: Dataset. DOI: https://doi.org/10.22002/nz145-65h12
